# Effects of Game Outcomes and Status Instability on Spectators’ Status Consumption: The Moderating Role of Implicit Team Identification

**DOI:** 10.3389/fpsyg.2022.819644

**Published:** 2022-02-01

**Authors:** Yonghwan Chang, Daniel L. Wann

**Affiliations:** ^1^Department of Sport Management, University of Florida, Gainesville, FL, United States; ^2^Department of Psychology, College of Humanities and Fine Arts, Murray State University, Murray, KY, United States

**Keywords:** biosocial theory of status, status instability, spectator sports, outcome uncertainty, sport consumer

## Abstract

This study explores the interaction effects of game outcomes and status instability and the moderating role of implicit team identification on spectators’ status-seeking behavior (the pursuit and preservation of social status). The current study seeks to contribute to the existing consumer behavior and spectatorship literature by examining the counterintuitive outcomes of winner–loser effects through the application of the biosocial theory of status. In an online experiment, NFL fans’ retrospective spectating experiences were captured and manipulated. This experiment used a 2 (game outcome: victory vs. loss) × 2 (status instability: decisive vs. close) × 2 (iTeam ID: high vs. low) between-subjects design. The findings indicated that decisive victories and close losses positively influenced spectators’ future attendance as well as their intention to purchase luxury suites and merchandise featuring images of the team mascot. Conversely, decisive losses and close victories had a negative influence. Additionally, the more spectators implicitly identified with a particular team, the more they exhibited status-seeking behavior; even close victories positively influenced the outcomes. By applying a nascent theoretical approach in the field of consumer behavior (the hormonal account), our results provide fresh insight into explaining spectators’ status-seeking behavior. Also, the findings identify specific conditions in which spectators’ status-seeking behavior is enhanced, thus suggesting ways for managers to strategically allocate their resources.

## Introduction

Consumer psychologists (e.g., Griskevicius and Kenrick, 2013; Saad, 2013) generally suggest that two distinctive motives work in tandem to form consumption behavior: proximate and ultimate motives. In brief, proximate motives are concerned with the antecedents and outcomes of consumer behavior, whereas ultimate motives investigate the biological roots and adaptive mechanism of such behavior (Saad, 2013); that is, the proximate motives explore *what* made people do a particular behavior, while the ultimate motives elucidate *why* such behavior is chosen and favored by individuals (Scott-Phillips et al., 2011).

In recent years, scholars have increasingly suggested that fundamental motives, as the evolutionary roots of human decision-making, have the potential to guide consumption behavior (Durante et al., 2011; Stanton, 2017). In particular, in the field of consumer behavior research, contemporary studies have paid attention to the fundamental motive of desire for both status and its stability (Griskevicius and Kenrick, 2013). Status refers to the respect and voluntary deference given to individuals by others in their surroundings (Mazzocco et al., 2012). Research suggests that individuals have a fundamental desire to be associated with others, and at the same time, they seek to obtain higher social ranks against others (Baumeister and Leary, 1995; Ligneul et al., 2016). Because humans are a social species, individuals who belong to a higher social status are likely to have easier access to material resources and/or greater societal influence in addition to exhibiting better psychophysiological health outcomes, compared to those who belong to a lower social status (Mazur, 1985; Mazur and Booth, 1998).

In marketing and consumer behavior literature, status-seeking behavior generally refers to individuals’ tendencies to seek out goods and services for the social status, standing, and recognition they confer^1^ (Eastman et al., 1999; Decrop and Derbaix, 2010). Among many options to reach a higher social status, watching sports may function as a way for spectators to attain such status (Decrop and Derbaix, 2010; Geniole et al., 2017; Burk et al., 2019). Given that spectators often perceive the victories and losses of teams they support as their own (Madrigal, 2008), a team’s victories can convey important symbolic meanings, such as a higher social status (Decrop and Derbaix, 2010). Thus, fans are more likely to continue watching and supporting their team when the team has outstanding records to enhance their self-esteem (Wann, 1995), feel positive emotions, such as pride (Mahony et al., 2002), and satisfy their need for success (Trail and James, 2001; Mahony et al., 2002; Robinson and Trail, 2005).

Also, according to the biosocial theory of status, the pursuit and preservation of social status is a basic characteristic of human nature (Mazur and Booth, 1998). Based on this theory, recent studies have suggested that the types of behavior in which consumers engage and the choices they make as consumers in their daily lives might be originally guided by internal cues, such as hormones (Durante et al., 2011; Saad, 2013; Durante et al., 2019). In particular, testosterone, which is a steroid hormone, likely serves as an internal cue that guides the fundamental mechanism behind these real-world consumer decisions (Griskevicius and Kenrick, 2013). When applying the biosocial theory of status to a sports context, changes in levels of testosterone correspond to outcomes in a competition (Archer, 2006; Geniole et al., 2017). However, because sport spectatorship is often impacted by the dynamics of a game’s processes, the extent to which competition results are decisive or close could influence the predictive potential of the biosocial theory of status (Geniole et al., 2017).

More specifically, according to the status instability hypothesis within the biosocial theory of status (Zilioli et al., 2014), in stable conditions (i.e., those in which outcomes are certain), experiencing victories is positively associated with testosterone concentration. In the case of close victories, however, tentative high status (i.e., a close victory) is likely to hinder the increase of testosterone because a high-status position is narrowly achieved (Archer, 2006; Geniole et al., 2017). On the other hand, achieving an unstable lower dominance ranking (i.e., a close loss) has counterintuitively been found to increase levels of testosterone, which in turn boosts consumers’ status-seeking behavior (Geniole et al., 2017). Thus, consideration of the status instability concept deems imperative in that tentative dominance ranking (i.e., close victory and close loss) is likely to moderate the relationship between game outcome and status-seeking behavior.

In addition to the influences that game results and game processes exert on the spectator, individual characteristics may also affect levels of testosterone. Casto et al. (2020) have shown that trait dominance (i.e., dominance motivation) to attain a higher status may boost levels of testosterone during competition. Given that dominance motivation reflects individuals’ fundamental preference for inequality and unbalanced power distribution between social groups (Ligneul et al., 2016), dominance motivation may be strongly associated with the concept of team identification (team ID) in the context of spectator sport. Further, given that dominance motivation is inherently implicit, unconscious, and shaped through cumulative socialization processes (McClelland et al., 1989; Schultheiss et al., 2005), we also predict that implicitly formed identification with teams (implicit team identification, iTeam ID; Chang et al., 2018) may be largely associated with dominance motivation. Based on this understanding, we attempt to examine (1) the effects of game outcome in conjunction with status instability and (2) the moderating role of iTeam ID on spectators’ status-seeking behavior. The current study contributes to the existing consumer behavior and spectatorship literature by illuminating the seeming paradox (i.e., reversed winner–loser effects due to status instability) of game outcomes through the application of the hormonal account.

## Theoretical Background and Hypotheses Development

### Biosocial Theory of Status and Game Outcomes

The major tenet of the biosocial theory of status is that winning a competition results in an increase in the level of testosterone, whereas losing a competition produces no such increase (Geniole et al., 2017). That is, winning, rather than losing, is likely to result in an increase in the level of testosterone. This hormonal fluctuation has been found to operate as cues in predicting a variety of consumption-related behaviors (Stanton, 2017). In particular, recent consumer behavior research has suggested that hormones, especially testosterone, serve as an internal cue in predicting real-life consumption choices (Geniole et al., 2017; Durante et al., 2019). Specifically, if one’s level of testosterone increases (as an internal cue), an individual may seek a higher social status as one of the fundamental human motives (i.e., attaining status) is activated (Griskevicius and Kenrick, 2013). Thus, an individual who won a competition not only feels higher status because of victory (Bernhardt et al., 1998), but also desires continued success in subsequent competition as a result of increased levels of testosterone (Zilioli and Watson, 2014; Casto et al., 2020).

The biosocial theory of status has garnered increased empirical support across a wide range of sport consumption contexts. For example, in a meta-analytic study focused on effects of competition outcome on testosterone in participants, winners showed larger pre- to post-competition increases in testosterone than losers, whereas the losers displayed decreased levels of testosterone (Archer, 2006; Geniole et al., 2017). Similar results were found in the context of spectator sports (e.g., Geniole et al., 2017; Burk et al., 2019). For instance, fans who rooted for the winning team when watching a World Cup match exhibited an increase in testosterone and status-seeking behavior after the match, relative to spectators who favored the losing team (Bernhardt et al., 1998). Pound et al. (2009) also found that spectators who were allocated to a “winning” condition of wrestling matches showed increased levels of testosterone; on the other hand, in spectators assigned to a “losing” condition, testosterone remained unchanged. Further, Casto et al. (2020) demonstrated that testosterone reactivity in response to game outcomes did not significantly differ by sex (even though men tend to show higher levels of testosterone).

In line with the biosocial theory of status, an extensive amount of spectatorship research has also suggested that victories often elicit positive emotions (e.g., happiness and pride; Madrigal and Chen, 2008; Decrop and Derbaix, 2010; Jang et al., 2017; see Wann and James, 2019, for a review) and pose significant implications for fans (e.g., vicarious achievement; Wann, 1995; Trail and James, 2001; Mahony et al., 2002; Robinson and Trail, 2005; Kim and Trail, 2010). Further, the favorable emotional experiences and implications conveyed from victories then lead to increased self-esteem, repeat patronage (Trail et al., 2005; Funk and James, 2006), luxury suites purchase behavior (Shapiro et al., 2012), consumption satisfaction (Moreno et al., 2015), willingness to pay premium (Kaiser et al., 2019), and BIRGing behavior (Madrigal and Chen, 2008).

### Conjunctional Role of Status Instability

The biosocial theory of status may be useful in understanding dichotomous game outcome effects in sports spectatorship. However, scholars must consider how this simple understanding of game outcome might not be able to fully elucidate the entire set of spectating experiences. For example, it has been suggested to consider how viewers’ experiences respond to or are affected by the dynamics of game processes (Jang et al., 2017; Chang, 2019; Chang and Inoue, 2021), in-game suspense (Bryant et al., 1994; Su-lin et al., 1997), and the element of surprise (McGraw et al., 2005). These dynamics merit a more nuanced analysis considering that fans’ spectating experiences, such as their game satisfaction and their intention to revisit another competition, are often influenced by such game dynamics. Therefore, given that limitation of the biosocial theory of status, the implications of the status instability concept (Welker et al., 2015) might represent a valuable approach to better understand the dynamics of the game process in conjunction with game outcomes.

The status instability concept included in the biosocial theory of status (Mazur, 1985; Casto and Edwards, 2016) asserts that the extent to which game outcomes are decisive or close could alter the predictions of the outcome effects of the competition. Specifically, in stable environments in which outcomes are certain, experiencing victories helps produce testosterone, and higher concentrations of this hormone have been positively associated with the subjective enjoyment of a competition (Welker et al., 2015). The meta-analytic review of winner–loser effects (Archer, 2006; Geniole and Carré, 2018) also suggested that when both men and women experience a victory in a team competition, they exhibit an increase in their levels of testosterone in comparison to those on the losing team. Additionally, the increased levels of testosterone in stable and secure high-status positions (i.e., decisive victories) have been found to result in dominance-seeking and competitive behavior as well as further attempts to gain higher status (Casto et al., 2020).

Understanding how spectators interpret the game outcomes of sporting events in conjunction with their status instability would be critical because it may provide valuable information of the types and specific attributes of products fans would attend to (as a means to comply with their augmented/attenuated status-seeking desire). In this respect, according to Veblen’s theory of conspicuous consumption, individuals tend to consume highly conspicuous products to satisfy their desire of signaling and enhancing social status (Bagwell and Bernheim, 1996). Gao et al. (2016) and Zheng et al. (2018) also suggest that status seekers often consider how much a product could symbolically pose an advantage for them, such as a higher social rank. As a result, consumers who desire social status tend to purchase luxury and premium products to gain recognition from others (Gao et al., 2016; Roux et al., 2017). In recent years, many sport venues have created new premium seating portfolios that include more affordable options for individual and budget-conscious markets, thus making premium seating options more accessible to a diverse audience of sport consumers (Sunnucks, 2018).

Rucker and Galinsky (2009) also suggested that status seekers tend to prefer visibly displayed and bigger logos, compared to their counterparts. Similarly, dominance motivation manifested in status-seeking behavior is often characterized as pursuing future competition opportunities to assert comparative dominance (Bernhardt et al., 1998; Casto et al., 2020) as well as pursuing a large (vs. small) figure of the consumer representing a feeling of power or being in control (as opposed to being controlled or submissive; Morris, 1995). As such, various forms of status-seeking tendencies would emerge in the given context, including: (1) status consumption, (2) intention to attend future games, (3) intention to purchase premium seats for future games, and (4) intention to purchase merchandise featuring an image of the team mascot (MERCH).

*Hypothesis 1:* A “decisive victory” *positively* influences spectators’ (a) status consumption, (b) intention to attend future games, (c) intention to purchase premium seats for future games, and (d) intention to purchase MERCH.

*Hypothesis 2:* A “decisive loss” *negatively* influences spectators’ (a) status consumption, (b) intention to attend future games, (c) intention to purchase premium seats for future games, and (d) intention to purchase MERCH.

On the other hand, status instability may induce contradictory winner–loser effects. In the case of close victories, for example, the winners’ higher status position is unstable, given that this higher status was narrowly attained (Mehta et al., 2008). This insecure sense of status (i.e., social status instability) is likely to hinder concentrations of testosterone (Archer, 2006; Geniole et al., 2017), which in turn, encourages individuals to actively avoid further competitions and other status-seeking attempts as a means to protect their current higher, yet still susceptible, social standing (Mehta et al., 2008). As such, close victories engender individuals to participate in fewer follow-up competitions while engaging in more status-maintaining behavior (Vermeer et al., 2020). Inversely, attaining an unstable lower dominance position (i.e., a close loss) has paradoxically been found to increase levels of testosterone (Zilioli et al., 2014; Wu et al., 2017) and status-seeking tendencies (Oliveira et al., 2009; Zilioli and Watson, 2014). This phenomenon is a byproduct of unexpected and surprising defeats, which produce an acute concentration of testosterone, encouraging status-seeking behaviors so that they can recover their status through a future competition (Vermeer et al., 2020). Based on this understanding, we formulated the following hypotheses.

*Hypothesis 3*: A “close victory” *negatively* influences spectators’ (a) status consumption, (b) intention to attend future games, (c) intention to purchase premium seats for future games, and (d) intention to purchase MERCH.

*Hypothesis 4*: A “close loss” *positively* influences spectators’ (a) status consumption, (b) intention to attend future games, (c) intention to purchase premium seats for future games, and (d) intention to purchase MERCH.

### Dominance Motivation and Implicit Team ID

Changes in the concentration of testosterone, which is represented through individuals’ status-seeking behavior, can also vary as a function of individual characteristics. In particular, Casto et al. (2020) suggested that dominance motivation for attaining high-status positions boosts the level of testosterone. Also, dominance motivation encourages an individual to recognize the disparity between in-group and out-groups and to desire an advantageous position in comparison to out-groups (Carlson et al., 2009; Ligneul et al., 2016). In the context of spectator sports, the concept of team identification (team ID) may be strongly and positively associated with the individual characteristic of dominance motivation. Team ID refers to “the extent to which individuals perceive themselves as fans of the team, are involved with the team, are concerned with the team’s performance, and view the team as a representation of themselves” (Branscombe and Wann, 1992, p. 1017).

Social identity theory has been widely applied to a variety of sport settings, including the examination of fan’s identification (ID) with sport teams (Boen et al., 2020). In the view of social identity theory, ID is a cognitive state in which a person comes to view him or herself as a member of a social entity (Bhattacharya et al., 1995) because of an overlap between their self-schema and the entity’s schema (Tajfel, 1978; Bergami and Bagozzi, 2000). Further, the theory asserts that individuals adapt their attitude and behavior to be of help to the in-group, whereas they take an unfavorable attitude to the out-group (Lock and Heere, 2017), and accordingly, group ID is based on the distinction between groups (i.e., in-group vs. out-group) and their power imbalance (Carlson et al., 2009). Also, individuals tend to join a group to enhance their status and reputation (Heere and James, 2007; Lock and Heere, 2017), and thus, spectators’ in-group ID with teams would be closely associated with their desire to dominate and be superior to out-groups. Similarly, sport business scholars have shown that fans with high team ID are more likely to engage in BIRGing behavior than those with low team ID because BIRGing helps realize fans’ desire to associate with successful others to enhance their self-esteem and status afforded by others (Wann, 1995; Trail and James, 2001; Mahony et al., 2002; Robinson and Trail, 2005). Also, Carlson et al. (2009) found that spectators with higher levels of ID purchase merchandise as a way to demonstrate their prestige and affiliation with a team (as a means of status-enhancement).

Although the validity and explanatory power of the existing team ID concept have been firmly established in spectatorship research, an increasing number of studies have suggested the prevalent role of the implicit aspect of identification (Alter and Oppenheimer, 2009; Greenwald et al., 2009; Chang et al., 2018). For example, McClelland et al. (1989) suggested that behavior is regulated by two independently operating systems—the explicit and implicit motivational systems. According to Schultheiss et al. (2005), the explicit motivational system is comprised of conscious cognitions and thus accounts for an individual’s explicit beliefs and goals shaped through effortful and systematic information processing. On the other hand, the implicit motivational system reflects unconscious, heuristic, and spontaneous preferences for activities that provide enjoyable affective incentives (Bettiga et al., 2017). Based on this theoretical background of implicit and explicit identification, in the context of spectator sport, Chang et al. (2018, p. 335) defined implicit team identification (iTeam ID) as “individuals’ stable representation of self-concept with a particular team, which is shaped without conscious awareness but that stems from long-term membership experience with the team.”

Further, McClelland et al. (1989) also suggested that dominance motivation is inherently implicit in the sense that: (i) individuals with high dominance motivation are not born with this motivation but acquire it through cumulative socialization experiences; (ii) highly dominance-motivated individuals often show consistent behavioral outcomes across environmental changes because they have learned that motivated dominance leads to pleasant consequences; and thus (iii) dominance motivation functions outside of conscious awareness and is rarely correlated with questionnaire-based measure of self-attributed dominance. Similarly, biological and hormonal responses are generally considered to be automatic, unconscious processes that occur without deliberate judgment (Griskevicius and Kenrick, 2013; Geniole et al., 2017). Accordingly, instead of consciously constructed identification, it would be beneficial to consider implicitly activated and operated identification to elucidate unconscious psychological features (i.e., dominance motivation) and automatic biological factors (i.e., testosterone). As such, we posit that iTeam ID may largely be associated with dominance motivation.

*Hypothesis 5*: Implicit team identification (iTeam ID) interacts with the game outcome and status instability effects in signifying spectators’ (a) status consumption, (b) intention to attend future games, (c) intention to purchase premium seats for future games, and (d) intention to purchase MERCH.

## Materials and Methods

### Design and Participants

The purpose of the current experiment was to examine whether the interaction between game outcome and status instability in conjunction with iTeam ID leads to a greater likelihood of status-seeking behavior. This experiment used a 2 (game outcome: victory vs. loss) × 2 (status instability: decisive vs. close) × 2 (iTeam ID: high vs. low) between-subjects design. With respect to the status instability manipulation, by following the existing guidelines (e.g., Cornil and Chandon, 2013), four games of a professional American football team were identified and classified as a close victory (second game, +2 points), close loss (10th game, −5 point), decisive loss (third game, −21 points), and decisive victory (14th game, +28 points). We recruited consumer panels through Qualtrics.^2^ Pre-screening qualifications included fans who: (1) report a favor with the target team and (2) attended at least one of the team’s four games. From the initial pool of spectators, 521 of those accepted the online survey invitation distributed about 2 months later given the target team’s completion of the regular season and shared their retrospective spectating experiences.

### Procedure

Once participants accepted the invitation, the Team ID IAT as a measure of iTeam ID (Chang et al., 2018) was prompted by utilizing the INQUISIT online software (Inquisit 5 Web)^3^; by following the existing guidelines, participants sorted the four target stimuli (i.e., four different images of the target team logo and mascot) into either the “Us” or “Them” in each main stage, respectively. For example, if participants were asked to pair the target stimuli with the “Us” category in the second stage, they were required to match the target stimuli to the “Them” category in the third stage. The order of the two stages was randomly counterbalanced across participants to prevent order effects.

Once participants completed the Team ID IAT procedure, they began the retrospective spectating experience task. As part of this task, participants were asked to recall one of the four games they had attended and write about their spectating experience of the game processes and outcomes. The experience sampling approach, including the retrospective spectating experience task, utilizes episodic and experiential responses by obtaining participants’ reports indicating their thoughts, behavior, and/or any physiological markers at the time of the assessment (Larson and Csikszentmihalyi, 1983). In this task, they were asked to remember the game they attended (most vivid game if attended multiple events) and write about their spectating experience with respect to the extent to which the outcome of the game was close, uncertain, and dramatic. To aid their retrospective memory of the selected game and to provide a visual supplement, a 1-min length video clip summarizing the game was extracted from YouTube.com and shown to the participants. Participants were then asked to respond to the measure of outcome uncertainty (“the game outcome was”: 1 = *uncertain*, 7 = *certain*; 1 = *unclear*, 7 = *clear*; 1 = *close*, 7 = *decisive*; and 1 = *surprising*, 7 = *not surprising*), developed based on the existing concept of (perceived) uncertainty (Cornil and Chandon, 2013; Hogan et al., 2017; Shen et al., 2019).

Next, participants responded to the proxy measures of status-seeking behavior including: (1) status consumption, (2) intention to attend future games, (3) intention to purchase premium seats for future games, and (4) intention to purchase merchandise featuring an image of the team mascot (MERCH). Status consumption was captured by using the randomly distributed five-item scale (e.g., “*pay more for a product if it had status*”: 1 = *strongly disagree*, 7 = *strongly agree*; Eastman et al., 1999). Future attendance (“*intention to attend games that would bring a similar experience*”; Fink et al., 2004) was measured using 3 seven-point scale formats (1 = *very unlikely*, 7 = *very likely*, 1 = *definitely would not*, 7 = *definitely*; and 1 = *strongly uninterested*, 7 = *strongly interested*). Both the intention to purchase premium seating (“*intention to purchase a premium seating option for the team’s future games*”) and to purchase MERCH (*“intention to purchase the team’s logo/mascot printed apparel*”) were measured by using pictorial measures along with a single seven-point scale format (1 = *very uninterested*, 7 = *very interested*), respectively.

## Results

### Preliminary Analyses

The initial 521 responses were screened, which resulted in eliminating 114 incomplete responses and 28 responses that failed the two attention questions (Downs et al., 2010), leaving a final sample of 379. Data were analyzed with R 4.0.5 (R Development Core Team, 2021). In terms of sample characteristics, nearly 69% of the participants were male (*n* = 262) while 31% were female (*n* = 117). The average age was 36.7 years old (SD = 10.82). The majority had a college- or graduate-level education (*n* = 299, 79%), and 82% of the respondents were Caucasian (*n* = 310), followed by African Americans (11%, *n* = 41), and other ethnic groups (7%). With respect to annual income level, about 41.7% of the participants (*n* = 158) indicated an annual salary of lower than $24,999, 38.4% (*n* = 145) indicated between $25,000 and $49,999, 17.8% (*n* = 67) between $50,000 and $99,000, and 2.1% (*n* = 9) indicated annual income greater than $100,000. The status instability manipulations were checked through the survey measures of outcome uncertainty. The four items were averaged to designate a single measure (*α* = 0.73). The two conditions of close victories (*M* = 2.71) and close losses (*M* = 2.43) revealed significantly different perceptions of uncertainty compared to the two conditions of decisive losses (*M* = 4.01) and decisive victories (*M* = 4.28), respectively; *F*(3, 375) = 94.82, *p* < 0.001.

With respect to iTeam ID as assessed through the Team ID IAT, we followed the existing scoring algorithm, that is, calculating the mean response latency difference between the two main stages (Bluemke and Friese, 2008; Chang et al., 2018). Specifically, we estimated the iTeam ID score by subtracting the mean response latency in the target team—“Us” stage from the target team—“Them” stage, and then, the latencies were transformed to *z*-scores; the score of iTeam ID was in the range of 1 to −1. Thus (same as the traditional IAT), the higher the mean latency differences between the two stages, the higher an individual’s implicit identification with the target team. Also, by following the existing guidelines (Bluemke and Friese, 2008), latencies below 300 milliseconds (ms) and above 3,000 ms were re-coded to their respective scores, and the first trial of each block was removed to achieve internal reliability (*α* = 0.86) as well as to reduce error rates (4.12%). The iTeam ID scores were not significantly different across the four game situations [*F*(3, 375) = 2.68, *p* = 0.11]. We averaged the multiple items measuring status consumption (*α* = 0.83) and intention to attend future games (*α* = 0.94) to form a composite measure, respectively.

### Main Analyses

#### Test of Hypothesis 1, 2, 3, and 4

A series of 2 (game outcome: victory vs. loss) × 2 (status instability: decisive vs. close) between-subjects ANOVAs were conducted along with follow-up univariate analyses.^4^ The results revealed significant main effects of game outcome on all of the DVs {status consumption [*F*(1, 375) = 4.26, $\eta_{p}^{2}$ = 0.11, *p* = 0.04; *M*_victory_ = 0.08 and *M*_loss_ = −0.11], intention to purchase premium seats for future games [*F*(1, 375) = 6.73, $\eta_{p}^{2}$ = 0.17, *p* = 0.009; *M*_victory_ = 0.11 and *M*_loss_ = −0.15], and intention to purchase MERCH [*F*(1, 375) = 8.46, $\eta_{p}^{2}$ = 0.19, *p* = 0.004; *M*_victory_ = 0.12 and *M*_loss_ = −0.16]}. On the other hand, status instability had non-significant main effects on status consumption [*F*(1, 375) = 2.34, *p* = 0.13; *M*_Decisive_ = 0.07 and *M*_Close_ = −0.06].

However, status instability significantly interacted with game outcome. Specifically, a decisive victory, compared to a decisive loss, produced significantly greater levels of status consumption (*M*_decisive loss_ = −0.57 and *M*_decisive victory_ = 0.53, $\eta_{p}^{2}$ = 0.24, *p <* 0.001) as well as greater intentions to attend future games (*M*_decisive loss_ = −0.53 and *M*_decisive victory_ = 0.19, $\eta_{p}^{2}$ = 0.21, *p < 0*.001), to purchase premium seats (*M*_decisive loss_ = −0.25 and *M*_decisive victory_ = 0.39, $\eta_{p}^{2}$ = 0.20, *p < 0*.001), and to purchase MERCH (*M*_decisive loss_ = −0.07 and *M*_decisive victory_ = 0.23, $\eta_{p}^{2}$ = 0.19, *p < 0*.001). These results supported the hypothesis that decisive victories positively influence spectators’ status-seeking behavior (*H1a*, *H1b*, *H1c*, and *H1d*), while decisive losses negatively influence their status-seeking behavior (*H2a*, *H2b*, *H2c*, and *H2d*).

Interestingly, status instability (i.e., the “close” conditions) reversed this straightforward tendency of the effects of victories and losses. That is, compared to a close victory, a close loss produced significantly greater levels of status consumption (*M*_close loss_ = 0.30 and *M*_close victory_ = −0.35, $\eta_{p}^{2}$ = 0.20, *p < 0*.001) as well as greater intentions to attend future games (*M*_close loss_ = 0.36 and *M*_close victory_ = 0.03, $\eta_{p}^{2}$ = 0.12, *p = 0*.02). Also, a close loss led to greater intentions to purchase MERCH (*M*_close loss_ = 0.32 and *M*_close victory_ = 0.02, $\eta_{p}^{2}$ = 0.13, *p* = 0.01). These results provide empirical support for the assumptions that close victories negatively influence spectators’ status-seeking behavior (*H3a*, *H3b*, and *H3d*), while close losses positively influence their status-seeking behavior (*H4a*, *H4b*, and *H4d*). However, the results rejected *H3c* and *H4c*. Tables 1 and 2 summarize the results.

**Table 1:** A summary of ANOVA results.

Variables		*F*-statistics for the dependent variables (Sig)			
		Status consumption	Future games	Premium seats	MERCH
Game outcome		4.26 (0.04)	6.99 (0.009)	6.73 (0.009)	8.46 (0.004)
Status instability		2.34 (0.13)	4.34 (0.04)	5.60 (0.02)	10.16 (0.002)
Game outcome × Status instability		87.34 (<0.001)	18.74 (<0.001)	12.92 (<0.001)	39.99 (<0.001)
		**M (SE) for the dependent variables**			
*Game outcome*	*Status instability*	*Status consumption*	*Future games*	*Premium seats*	*MERCH*
Victory	Decisive	0.53 (0.19)^a^	0.19 (0.23)^a^	0.39 (0.31)^a^	0.23 (0.28)^a^
	Close	−0.35 (0.23)	0.03 (0.23)	−0.15 (0.28)	0.02 (0.27)
Loss	Decisive	−0.57 (0.17)	−0.53 (0.21)	−0.25 (0.29)	−0.07 (0.14)
	Close	0.30 (0.24)^b^	0.36 (0.19)^b^	−0.06 (0.21)	0.32 (0.26)^b^

The numbers of participants for each cell include 107_Decisive Victory_, 112_Close Victory_, 76_Decisive Loss_, and 84_Close Loss_.

a Significantly greater than the Decisive Loss condition.

b Significantly greater than the Close Victory condition.

**Table 2:** Standardized means (*M*), standard deviations (SD), unstandardized path coefficients (*B*), and standard errors (SE).

Conditions	DVs	*M*	SD	*B*	SE	*p*
Decisive Victory	Status consumption	0.29	0.07	1.25^***^	0.24	<0.001
	Future games	0.12	0.11	1.75^***^	0.30	<0.001
	Premium seats	0.04	0.13	1.13^*^	0.35	0.01
	MERCH	0.13	0.29	0.33^*^	0.51	0.03
Decisive Loss	Status consumption	−0.17	0.27	−0.15	0.35	0.66
	Future games	−0.33	0.24	0.12	0.45	0.72
	Premium seats	−0.15	0.21	0.09	0.37	0.79
	MERCH	−0.27	0.13	−1.31^***^	0.38	<0.001
Close Victory	Status consumption	−0.01	0.21	0.98	0.54	0.07
	Future games	0.06	0.19	1.26^*^	0.54	0.02
	Premium seats	−0.03	0.23	1.39^*^	0.55	0.01
	MERCH	0.01	0.28	1.07^*^	0.49	0.03
Close Loss	Status consumption	0.21	0.23	0.98^*^	0.48	0.03
	Future games	0.08	0.17	1.56^**^	0.52	0.002
	Premium seats	0.09	0.31	1.19^**^	0.44	0.004
	MERCH	0.11	0.16	0.99^*^	0.52	0.04

* *p* < 0.05;

** *p* < 0.01;

*** *p* < 0.001.

#### Test of Hypothesis 5

Generalized linear models (GLM) were used to examine a linear regression model for the four continuous response variables given the continuous (iTeam ID) and categorical predictors [the four game situations; 2 (game outcome: victory vs. loss) × 2 (status instability: decisive vs. close)]. The results revealed that iTeam ID significantly influenced status consumption (*H5a*, *B* = 0.75, SE = 0.24, *t* = 3.15, *p* = 0.002), intention to attend future games (*H5b*, *B* = 1.09, SE = 0.23, *t* = 4.65, *p* < 0.001), intention to purchase premium seats for future games (*H5c*, *B* = 0.96, SE = 0.23, *t* = 4.08, *p* < 0.001), and intention to purchase MERCH (*H5d*, *B* = 1.07, SE = 0.38, *t* = 2.85, *p* = 0.005). Thus, the stronger the spectators’ iTeam ID, the greater their likelihood to seek a higher status.

**Figure 1:**
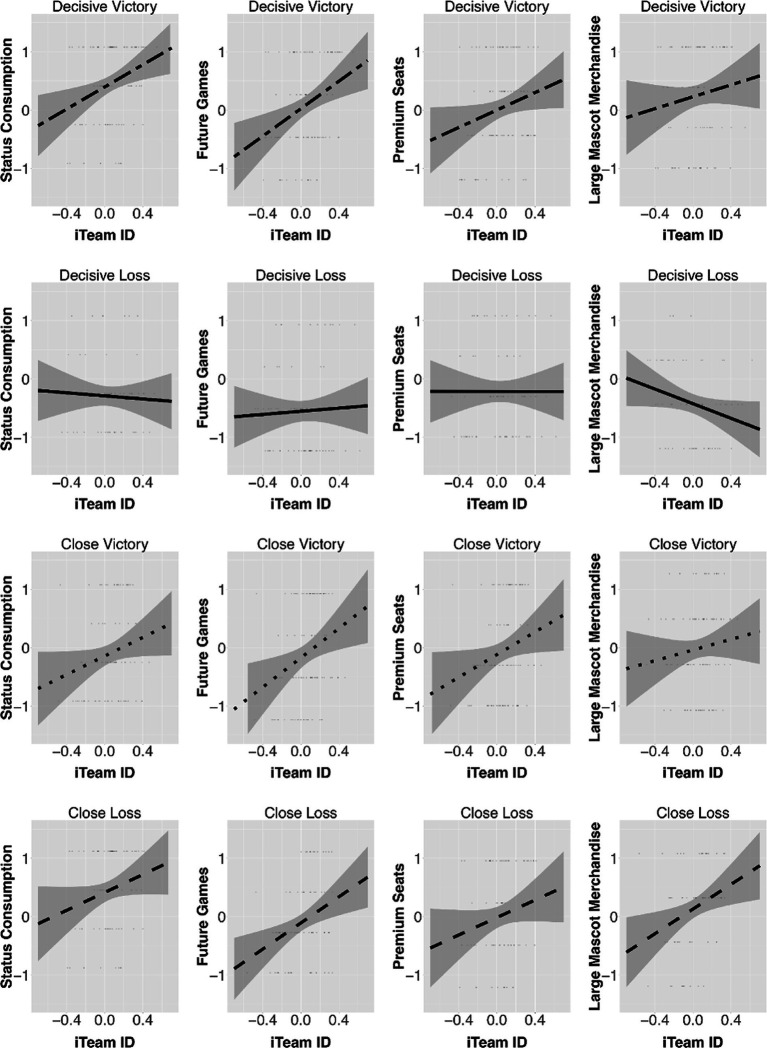
A summary of the generalized linear model results.

More specifically, the three conditions of a decisive victory, a close victory, and a close loss similarly interacted with iTeam ID to influence the DVs. For example, the stronger the participants’ iTeam ID, the higher their likelihood to seek a higher status (e.g., intention to purchase premium seats for future games: *B* = 1.39, SE = 0.55, *t* = 2.53, *p* = 0.01 in the close victory condition). However, the influence of iTeam ID on DVs in the decisive loss condition were either non-significant (e.g., intention to attend future games: *B* = 0.12, SE = 0.35, *t* = 0.36, *p* = 0.72) or negatively significant (e.g., MERCH: *B* = −1.31, SE = 0.38, *t* = −3.85, *p* < 0.001). Accordingly, even close victories (in addition to decisive victories and close losses) positively influenced spectators’ status-seeking behavior when their iTeam ID was high. Figure 1 summarizes the GLM results.

## Discussion

### Theoretical Implications

In the current work, we explored the interactions of game outcome and status instability effects on spectators’ status-seeking behavior in conjunction with iTeam ID. Based on empirical support, we delineated several important theoretical contributions to the literature. Most prominently, by applying a nascent theoretical approach in the field of consumer behavior (i.e., the hormonal account; Durante et al., 2019), our results provide fresh insight into explaining spectators’ status-seeking behavior. Existing sport consumer behavior research has primarily utilized proximate motives (Funk et al., 2012; Ciomaga, 2013) to account for what motivates people to engage in sport-related activity and how their cognitive needs and motivations are related to consumption behavior (e.g., McDonald et al., 2002; Funk and James, 2006; Funk, 2017). Although these proximate explanations have been widely employed [e.g., expectancy (dis)confirmation theory; Trail, 2018; social identity theory; Lock and Heere, 2017; psychological continuum model; Funk and James, 2001], scholars in psychology have increasingly suggested that fundamental motives, as the evolutionary roots of human decision-making, have the potential to complement proximate motives (Durante et al., 2011; Saad, 2013; Stanton, 2017). For example, Saad (2013, p. 366) points out that “fundamental motives complement the rigorous and sophisticated research streams that consumer scholars currently engage in, by offering explanations rooted in ultimate causation.” That is, it has been suggested to also consider the fundamental motives of a human being, which focuses on the unconscious psychological mechanism and hormone action, to have a thorough understanding of individuals’ decision-making process (Durante et al., 2011; Saad, 2013).

In response to the calls for more research on biological motives and organismic perspective in the realm of sport management (e.g., Funk et al., 2012), we have attempted to expand our understanding of why status-seeking behavior is adapted and pursued by spectators by applying the biosocial theory of status (i.e., the ultimate motives approach). Further, Funk (2017) suggested that sport consumer behavior research is predominantly relying on cross-sectional survey research to merely test the generalizability of the existing findings. We agree with Funk’s arguments that experimental methods should be more proactively pursued to examine the “theoretical causality” more precisely (Shadish et al., 2002); such efforts would help promote theory development and advance knowledge discovery in sport consumer behavior research. Accordingly, we have adapted an experimental approach to explore causality associated with such status-seeking tendency by identifying and examining the proxy indicators of behavioral outcomes given isolated specific game conditions. Further theoretical implications corresponding to the results of each experimental condition are discussed in the following sections.

#### Decisive Victories and Losses

The results revealed that, regardless of the level of iTeam ID, spectating experiences of decisive victories or losses enhance or weaken, respectively, spectators’ intention to attend future games and their status consumption (as manifested in their future intentions to purchase a luxury suite and MERCH). These results provide empirical support for the hormonal account of consumption behavior. That is, according to the biosocial theory of status (Casto and Edwards, 2016; Ligneul et al., 2016), testosterone changes caused by experiencing a victory or a loss operate as biological modulators of status-seeking behavior. Specifically, winning, as opposed to losing, enhances the concentration of testosterone, which serves as an internal cue triggering competitive and status-seeking behavior (Bernhardt et al., 1998; Oliveira et al., 2009; Geniole et al., 2017) and resulting in positive mental health consequences (Cohen et al., 2016; Snyder et al., 2016).

These straightforward results share similarities with existing findings in the sport business and marketing literature. Sport fans often perceive the achievement of teams they support (e.g., high team ID) as their own success (Madrigal, 2008). Therefore, victories lead to positive emotions of fans, such as happiness (Jang et al., 2017) and pride (Mahony et al., 2002; Decrop and Derbaix, 2010), in addition to the other significant implications it represents for spectators, such as experiencing vicarious success (Trail and James, 2001; Mahony et al., 2002) and increased self-esteem (Wann, 1995). In turn, these emotions and processes positively influence spectators’ subsequent consumption behavior as manifested through repeat patronage (Trail et al., 2005; Funk and James, 2006) and BIRGing behavior (Madrigal and Chen, 2008).

#### Close Victories

The results revealed how significant interactions among game outcome, iTeam ID, and status instability affect status-seeking behavior. That is, for spectators with lower iTeam ID, experiencing a close victory produced lower intentions of future attendance and premium seating purchases, and weaker preference for MERCH. Our results, therefore, would challenge past approaches taken in the majority of sport consumer behavior research (e.g., the winner–loser effects; Kim and Trail, 2010; Shapiro et al., 2012). From the perspective of fundamental motives, a close victory can be understood as an unstable and illegitimate high-status position; within social hierarchies, individuals often show risk aversion tendencies by actively avoiding further competition when their dominance and status are considered to be tentative (i.e., unstable hierarchy; Mehta et al., 2008; Zilioli and Watson, 2014). This tendency has been suggested to occur as a means for consumers to merely preserve their superior social position (Geniole et al., 2017); in other words, subsequent competitions after a close victory may lead fans to become more concerned about the possible loss of their current status rather than enjoying a status gain (Mehta et al., 2008). As such, the attainment of an unstable high-status position has been weakly associated with spectators’ levels of testosterone but positively associated with avoidance of further contests (Geniole et al., 2017), weakened status-seeking behavior (Oliveira et al., 2009), and feelings of anxiety and nervousness (Cohen et al., 2016) and depressive symptoms (Snyder et al., 2016). Similarly, in the realm of sport management, Bernache-Assollant and Chantal (2011) suggested that fans tend to be less likely to support a potential loser (even though the team is currently celebrating victories) as a way to guard their future social identity from prospective damages (i.e., the Cutting of Future Failure process, COFFing; Wann et al., 1995).

On the other hand, the results revealed that for spectators with higher iTeam ID, spectating experiences of a close victory (in addition to decisive victories and close losses) produced higher intentions of future game attendance and premium seating purchases and stronger preferences for MERCH. These findings are consistent with research conducted by Carlson et al. (2009); the results of their study revealed that spectators with higher levels of team ID positively influenced the amount of money individuals spent on team-related retail purchases. These results support that implicitly, unconsciously, and cumulatively shaped identification with a team (i.e., implicit team identification, iTeam ID) largely reflects the characteristics of dominance motivation. Studies on the biosocial theory of status have suggested that dominance-motivated individuals are prone to experience feelings of excitement and enthusiasm when they are confronted with a challenge (Mehta et al., 2008; Ligneul et al., 2016). Enhanced social status is not an end state nor is it an ultimate goal for dominance-motivated individuals; rather, as a compensation for performing a challenging task, consumers may perceive an enhanced level of social standing (McClelland et al., 1989). Hence, status-seeking behavior is instrumental in producing such natural incentives as favorable feelings of excitement/stimulation and experiences of unforced concentration/flow. Mehta et al. (2008) also suggested that dominance-motivated individuals often experience an intrinsic enjoyment without conscious awareness that upon attainment, reinforces individuals’ status-seeking behavior, thereby eliciting consistent hormonal responses and actions. This prediction stems from the notion that dominance motivation is shaped through cumulative socialization experiences and therefore functions outside of an individual’s conscious control and awareness (McClelland et al., 1989).

The cognitive appraisal theory of emotions applied to sport business and marketing studies (McGraw et al., 2005; Decrop and Derbaix, 2010; Moreno et al., 2015; Kim et al., 2017) aligns with this finding. Dominance motivation may be associated with the self-conscious emotions of pride and shame (Decrop and Derbaix, 2010). Pride arises when individuals feel respect and favorable attention given by others, while shame can be elicited by interacting with dominant others who act hostile and dismissive toward them. Spectators with higher ID with teams often experience either pride or shame corresponding to their preferred teams’ performance (Decrop and Derbaix, 2010; Partridge et al., 2010). Therefore, the level of dominance motivation is likely to differ based on the extent to which individuals identify with a team. People with high team ID have been found to express more proactive behaviors and greater reactivity when faced with dominance challenges (Wann and Branscombe, 1990; Mazzocco et al., 2012). These findings also align with expectancy (dis)confirmation theory (Madrigal, 1995), as people expressed more intense emotional response and greater behavioral intention when the expectation of the game outcome was disconfirmed (Trail and James, 2001; Trail, 2018). In addition, according to the positive view of stress (Wann, 1995; Trail and James, 2001) and studies on flow (Jackson and Csikszentmihalyi, 1999; Chang et al., 2018), the higher individuals’ knowledge, skills, and involvement, the more the concurrent task should be (emotionally) stressed to augment their intrinsic and long-lasting pleasure. Such natural incentives then contribute to spectators’ wellbeing and life satisfaction so as to encourage them to perform the same tasks repeatedly (Jackson and Csikszentmihalyi, 1999).

#### Close Losses

The attainment of high status through victories should increase testosterone (Geniole et al., 2017), status-seeking behavior (i.e., dominance-seeking tendency; Edwards and Casto, 2013), and mental health (Snyder et al., 2016) in comparison to attaining low status. However, interestingly, the results revealed that regardless of the level of iTeam ID, spectating experiences of close losses are positively associated with spectators’ intention to attend future games and status consumption displayed in premium seating purchase intention and preference for MERCH. The results, therefore, provide initial support for the implications of status instability. Given the perspective of fundamental motives, the status instability hypothesis posits that an unstable low social status (i.e., a close loss) exerts an increase in testosterone (Geniole et al., 2017), which encourages status-seeking tendencies (Mehta et al., 2008). This phenomenon occurs because, according to Oliveira et al. (2009), testosterone increases after the loss of status in an unstable social hierarchy could indicate preparatory reactions for future encounters of competitions (i.e., anticipatory rise in testosterone). This seeming paradox (i.e., an increase in testosterone for losers compared to winners) is particularly observable in those losers who experienced the temporal feelings of threat and anxiety (Zilioli et al., 2014). Similarly, Jordan et al. (2011) observed the reversed association between social power and competitive behaviors; people who assigned to unstable and less powerful social positions (e.g., close victory) showed more competitive behavior (when compared to the group with stable and powerful positions). Furthermore, when a person’s positive social identity is threatened (e.g., close loss), he or she may employ self-enhancement strategies, such as purchasing MERCH or premium seating, to regain positive social identity (Phua, 2010).

Research in marketing and sport business share similar results. For example, sport economists (Rottenberg, 1956; Ely et al., 2015) and consumer behavior researchers (Shen et al., 2019) have suggested that spectators’ consumption behavior has been found to increase corresponding to the uncertainty of expected game outcomes (or the extent to which the results of a game is unpredictable so as to make the game dramatic and close; Trail and James, 2001). Similarly, Campbell et al. (2004) and Wann and Branscombe (1990) concurrently argued that fans often applaud poor performances by their preferred teams (i.e., Bask In spite of Reflected Failure, BIRF; Campbell et al., 2004) as a means to preserve their team identity and legitimize their psychological exclusivity and distinction from others. Along the same lines, according to the compensatory behavior account, individuals tend to compensate for any external threats by exaggerating the equity of their groups (Giulianotti, 2002) or by over-consuming objects that symbolize their social identity (Mazzocco et al., 2012).

### Practical Implications

Understanding how spectators interpret the outcome and process of sporting events is important to sport marketers because it allows them to better understand ways in which their product is being consumed. In this respect, the findings from this research suggest specific conditions where spectators’ intentions for future attendance and desire for status consumption are enhanced. First, the results have implications for team and facility marketers. When the three conditions emerge (including decisive and close victories and close losses), managers should actively display their commercials and promote their luxury suites and MERCH in the local area and any other relevant target markets (Roux et al., 2017). Moreover, it may be useful to focus on the signaling effectiveness of status goods and services, which could be done by emphasizing the conspicuous value of the product (Gao et al., 2016). Additionally, an activated status motive has been suggested to make individuals less sensitive to product price since cheaper products are often considered to represent a lower status (Eastman et al., 1999). Accordingly, proactive marketing and promotion of premium quality products and team merchandise are recommended. On the other hand, in the case of decisive losses, marketers may consider promoting and advertising their team MERCH and limit their investment on the promotion of luxury and premium products. Reasonably priced, moderate quality products may be a more attractive option in this case.

Second (although pro-environmentalism specific factors were not examined in this study), the results may have implications for cause-related marketing given the wide-ranging applications of the hormonal account (Hand, 2020). More specifically, people often compete for prosocial reputations and images that help enhance their social status (e.g., competitive altruism; Mazzocco et al., 2012). That is, individuals often desire to be seen as social and environmentally friendly by spending more money on others rather than on themselves through which they can take advantage of a higher social status (Rucker and Galinsky, 2009; Hand, 2020). Hence, given the emergence of such events (decisive and close victories and close losses), proactive marketing and promotion to help alleviate their target markets’ hunger for status are recommended. Such efforts might then efficiently lead to an increase in spectators’ willingness to pay premiums for environmentally “green” and “socially responsible” products.

Third, the current study provides implications for policy makers and public health officials. Research has revealed that mental health benefits individuals in multiple ways. For example, mental health is related to greater self-control, higher levels of subjective wellbeing, better interpersonal relationships, an increase in life satisfaction, greater creativity, higher levels of work performance and satisfaction, and better physical health (Löwe et al., 2010; Cohen et al., 2016). Policy makers should attend to the condition of decisive losing. Losers in legitimate and stable positions often face psychological punishment from winners as well as from other frustrated losers (Jauernig et al., 2016). Consequently, proactive socially responsible initiatives, such as mental health treatment and depression prevention efforts should be conducted.

### Limitations and Future Suggestions

The following limitations and suggestions should be addressed in future studies. First, the utilization of retrospective spectating responses could be confounded by potential covariates (e.g., memory bias and dilution of verbatim traces of experience); in other words, participants’ inaccurate recollection of their experience might have distorted the results. Real-time measures or reducing the time gap between events and data collection would help resolve this issue. Second, response latency-based measures (including IAT) are one of the most frequently applied tools in accessing individuals’ fundamental and unconscious mind (Greenwald et al., 2009); nonetheless, the validity of the results should further be tested in future studies to firmly establish its conceptual/methodological rigorousness given that the Team ID IAT is a newly emerging tool in sport management research (e.g., testing nomological validity by associating perceived team ID with iTeam ID).

Third, given the utilization of the stratified sampling approach as well as the self-select manipulation of the game outcome and status instability factors, individual characteristics for each condition would be variant (e.g., age, gender, past spectatorship experiences, and explicit team identification), limiting the internal validity of the results. Future scholarship, therefore, should replicate the study by using broader and diverse contextual (e.g., NFL, World Cup, and other collegiate and professional sporting events) and sampling frames (e.g., fans who attended all game situations) given the study’s limited scope to a single football team as a target object. Similarly, conditioning multiple games for a single manipulation condition would also enhance the generalizability of the results as we only had a single game for each factorial condition.

Fourth, scholars in business and consumer behavior (e.g., Stanton, 2017; Durante et al., 2019) are increasingly suggesting that hormonal accounts would provide a fundamental understanding of human decision-making, and these accounts could be applicable to a variety of consumption contexts by utilizing proxy measures without direct measurement of hormones; nonetheless, future studies would adapt different types of status-seeking tendencies (e.g., temporal focus of emotions, such as feelings of pride) or simple forms of hormone measures (e.g., salivary testosterone) to further validate the results of the current study. Last, future studies should consider other potential covariates associated with in-game (e.g., the rivalry effect, the opponent team’s record, and supporting team’s performance) and individual characteristics (e.g., identity management and coping strategies; Bernache-Assollant and Chantal, 2011; coping strategies; Delia, 2019) that might have conjunctional influences on spectators’ psychological and behavioral consequences. The aforementioned elements might have prevented *H3c* and *H4c* to reach to a full significance and limit the validity of the results; however, at the same time, these findings potentially represent exciting opportunities for future scholarship.

## Data Availability Statement

The raw data supporting the conclusions of this article will be made available by the authors, without undue reservation.

## Ethics Statement

The studies involving human participants were reviewed and approved by University of Minnesota. Written informed consent for participation was not required for this study in accordance with the national legislation and the institutional requirements.

## Author Contributions

YC and DW contributed to conception and design of the study together. YC performed the statistical analysis and wrote the first draft of the manuscript. All authors contributed to the article and approved the submitted version.

## Conflict of Interest

The authors declare that the research was conducted in the absence of any commercial or financial relationships that could be construed as a potential conflict of interest.

## Publisher’s Note

All claims expressed in this article are solely those of the authors and do not necessarily represent those of their affiliated organizations, or those of the publisher, the editors and the reviewers. Any product that may be evaluated in this article, or claim that may be made by its manufacturer, is not guaranteed or endorsed by the publisher.

## Footnotes

^1^Eastman et al. (1999, p. 42) further conceptualizes status consumption as “*the motivational process by which individuals strive to improve their social standing through the conspicuous consumption of consumer products that confer and symbolize status both for the individual and surrounding significant others*.”

^2^ http://www.qualtrics.com/

^3^ https://www.millisecond.com

^4^We tested the assumptions for two-way ANOVA in R 4.0.5. The results revealed no obvious violations of the underlying assumptions. For instance, the residuals were normally distributed and equal variances could also be assumed for all DVs (e.g., Levene’s test; *p* = 0.68 for MERCH; Fox et al., 2021).
